# Co-designing transport models as a heuristic planning tool

**DOI:** 10.1098/rsta.2024.0110

**Published:** 2024-11-13

**Authors:** Tanvi Maheshwari, Pieter Fourie

**Affiliations:** ^1^ Department of Architecture, Monash University, Melbourne, Victoria 3145, Australia; ^2^ Graduate School of System Design and Management, Keio University, Hiyoshi, Kohoku-ku, Yokohama, Kanagawa, Japan; ^3^ Graduate School of Advanced Science and Engineering, Hiroshima University, Kagamiyama, Higashi-Hiroshima, Hiroshima 739-8527, Japan

**Keywords:** agent-based simulations, urban design, co-design, automated vehicles

## Abstract

Recently the transportation sector has witnessed several new technologically driven disruptions that have amplified the complexity of city planning and policymaking. Traditional well-established processes of decision-making in urban planning and transportation are proving insufficient to deal with this degree of complexity and uncertainty. This paper proposes an alternative approach, combining qualitative and normative urban design, with quantitative and predictive transport modelling. This requires urban designers and transport modellers to co-create goal-driven and agile transport models that act as a heuristic tool to guide planning decisions in early design stages. Heuristic modelling is informed by design optioning and vice versa in an iterative loop. A case study is presented to demonstrate how this approach is operationalized to study the impacts of automated vehicles on urban planning. Design workshops are used as a method to elicit responses from stakeholders, which are used to co-create the simulation models. This collaborative process grounds the research in real-world practice and enhances the communication of design proposals and research findings across disciplines. By integrating design thinking methods with agent-based transport simulations, this approach provides a better understanding of emergent effects in complex urban systems and improves stakeholder engagement in the planning process.

This article is part of the theme issue ‘Co-creating the future: participatory cities and digital governance’.

## 1. Introduction

In recent years, the transportation sector has seen several new technologically driven disruptions that have increased the complexity of city planning and policymaking. Prominent examples of such technologies include the rapid growth of ridesharing/pooling platforms and the prospect of fully automated vehicles (AVs) operating on urban streets. In the face of such disruptions, policymakers often struggle to keep up with their pace of expansion, for example, the lag in regulations and infrastructure provisions vis-à-vis the growth of platform-based ridesharing services in cities [1]. Conversely, they can be overzealous in accommodating new technologies, evident in how some cities rushed to develop plans and infrastructure for a fully AV-ready future, relying on over-optimistic predictions of AV technology deployment. Interest in AVs has waned since the pandemic, and expectations for their deployment and consumer market have become much more modest [2].

These examples illustrate the challenging environment that city planners operate in today. The increase in uncertainty—both through internal factors like technology readiness and public acceptance, and external factors such as natural disasters and global pandemics—combined with increased interdependence across scales and subsystems, has led to a high degree of complexity. Traditional decision-making processes in urban planning and transportation are proving insufficient to handle this degree of complexity and uncertainty.

This paper examines the shortcomings of the traditional process of decision-making in transport infrastructure design in the context of the rising complexities and uncertainties accompanying the ‘technological shift in transportation’ [3]. We propose an alternative approach, with iterative engagement between qualitative and normative urban design, and quantitative and predictive transport modelling. This approach requires urban designers and transport modellers to co-create goal-driven and agile transport models as a heuristic tool to guide planning decisions in the early stages. A case study is presented to demonstrate how this approach is applied to study the impacts of the deployment of AVs in Singapore.

## 2. Planning and decision-making in transportation

**Figure 1.**
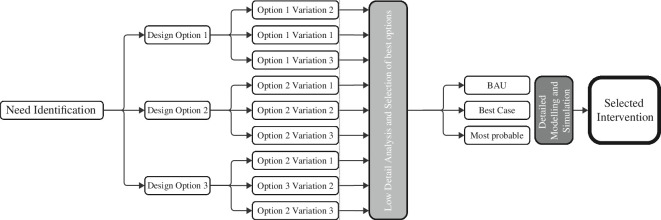
Typical planning and decision-making workflow for new transportation interventions.

Traditionally decision-making in planning for new transportation infrastructure follows a compartmentalized, linear process that relies heavily on predictive modelling, as shown in figure 1. First, the need for a new design or planning intervention is identified forming the design or planning brief. Next, a set of design options is developed and evaluated in consultation with stakeholders, based on established precedents, assumptions and guidelines. Following this, a small number of options are selected for a detailed appraisal exercise, conducted through predictive transport modelling and simulation. For decision-makers to make ‘rational’ and ‘auditable’ decisions, detailed quantitative models are central to the final appraisal process [4]. This paper argues that the technological shift in transportation calls for a rethinking of this process, to make it more transdisciplinary and iterative and less reliant on one-shot, predictive modelling, which often ends up as only an endorsement of foregone conclusions.

Built form and transport flows are deeply intertwined, yet often examined in isolation. The properties of transportation flows (volume, speed and technology) determine the specifications of the built form required to accommodate them. At the same time, it is well established in the literature that the design of the built form can influence travel behaviour and resulting transport flows [5–8]. However, a disciplinary divide exists between the provision of built form, which is situated in urban design and planning, and the prediction and assessment of transportation flows, which falls within the scope of transport planning and traffic engineering. Both disciplines use different methods and processes, often linked through a one-way compartmentalized ‘predict and provide’ approach [9,10].

Emerging new transport technologies are deeply interconnected and still in very early stages, making predictive models less reliable. Predictions tend to extrapolate past trends, which may be acceptable for short-term planning but can be problematic for long-term futures marked with rapid change and uncertainty [11–13]. Even as predictive transport models get more advanced, progressing from the aggregated top–down models to sophisticated bottom–up agent-based models and complex Land Use and Transport Interaction models, it is important to be reminded that since cities are complex systems, we cannot ‘predict the future’ for these systems [14].

Even though predictive models get progressively less reliable with increasing timeframes and uncertainties, they remain crucial to the decision-making process for new transportation interventions. They allow the decision maker to measure and evaluate the performance of various design options to arrive at a (seemingly) rational decision. A quantitative model helps build consensus by providing a rationale for large and expensive infrastructure investments, by implicitly shifting the burden of responsibility to an ‘impartial’ and ‘objective’ model. However, experience has shown that this line of thought is flawed.

Transport models can be riddled with biases by focusing only on the measurable variables. Quantifiable economic benefits are used as a justification for new infrastructure decisions, neglecting socio-political and cultural impacts that are harder to quantify [15,16]. For example, travel mode choice models are typically based on time and cost variables, with travellers choosing the shortest or cheapest route. However, in practice, travellers may opt for a longer route if it offers a more pleasant experience [17], through improvements in street design. While recent attempts have been made to include such sociocultural and behavioural nuances in transport models [18], these enhancements may also make the models overly complex and difficult to use for early stage analysis.

Predictive transport models have been criticized for their extensive time, data and cost requirements [19–22]. Large black-box models make it difficult to analyse the dynamics of planning interventions and their relationship to specific goals and sub-goals. Additionally, these models assume a consensus among all decision-makers regarding the goals and sub-goals the project needs to achieve, as well as the extent to which the interventions are effective in achieving them [23]. There is often as much uncertainty about goals as with planning interventions and outcomes [11,24]. However, the considerable cost and time investment can lock decision-makers into their initial position, making them reluctant to investigate alternatives especially if further exploration would challenge early assumptions and decisions. This is even more likely to occur in resource-limited environments with high population and economic pressures.

The issues and biases in transport modelling described here are often corrected using qualitative design methods in the decision-making process. Urban design is a future-oriented discipline [25] that is grounded in reality [26]. Although urban design is prescriptive, it is informed by predictions, and strategies such as visioning, scenario building and stakeholder engagement workshops [15,16,27,28] help connect predictions to the design process. However, the dominant methods and tools used in the design disciplines have limits when dealing with the increasing uncertainties and complexity of the transportation sector.

Urban design and planning methods tend to have a spatial bias and struggle to incorporate temporal dynamics. Some multi-disciplinary scholars have tried to integrate mobility flows and spatial planning in novel ways, such as the theory of ‘movement economies’ [29], operationalized through Space Syntax tools, and the ‘new mobilities paradigm’ [30] in geography. Although quantitative planning support systems have become more popular [31], space and time are rarely addressed together in urban design [5,8,13,32]. A two-dimensional plan is the favoured representation style, reinforced by the rise of GIS-based analytical tools [25,33]. These analyses abstract out complexities and fail to capture temporal change and emergence, which are fundamental properties of complex systems.

‘Models’ are used in both urban design and transport planning as a primary method for the analysis and synthesis of an abstracted reality. Yet how these models are defined and constructed differs dramatically between the two disciplines. The design process is iterative [34] and operates at a coarse level of detail in the early stages. Several design options are tested early and ‘best solutions’ are shortlisted based on network or GIS-based analysis, normative principles, stakeholder feedback, precedents, standards or simply intuition. A small selection of options is then handed over to the transport modeller for detailed simulations.

Transport modelling processes are comparatively less iterative. More information and parameters are progressively added to the model to improve accuracy, making predictive modelling expensive and time-consuming. As a result, early stage design options are rarely informed by transport models. This is problematic because large-scale transport models are costly to run; therefore, it is essential to ensure that the final set of options selected for detailed simulations is chosen carefully and rigorously. The early stages of design development could benefit from quantitative transport models that add the dimension of time and emergence to the assessment while reducing the risk of time and cost investment for detailed simulations by the transport modeller at a later stage.

The criticisms of predictive transport modelling in literature are primarily directed towards the so-called large-scale ‘consolidative modelling’ [35], which strives to add more detail to the models to get more accurate predictions. Since no amount of detail can provide complete validation in highly complex and uncertain systems, a model can only be expected to indicate the performance of an intervention under a set of predefined conditions. This view of an analytical model is what Portugali [36] describes as ‘heuristic planning tools’, Batty [20] as resembling ‘pedagogy’ in how they inform and extend our understanding, Guhathakurta [37] as a ‘narrative’ or ‘storytelling tool’, Perez *et al.* [38] as ‘mediating objects accompanying knowledge building and sharing’ and Bankes [35] as ‘exploratory modelling’.

This paper argues for integrating coarse low-detail transport simulation models as heuristic tools at early planning stages to inform decision-making. The following section details one case study project where agent-based transportation models were co-created with urban designers, transport modellers and policymakers, to be used as a heuristic planning tool.

## 3. Planning a city for shared automated vehicles (SAVs): a case study

Singapore’s long-term development goals of sustainable growth and high quality of life are being challenged as population growth continues to strain the already limited land resources. Automated and shared mobility are promising technologies to lower carbon and reduce spatial footprints. This case study is part of a research project investigating urban planning and design strategies to implement shared automated mobility to meet Singapore’s long-term sustainable development goals. The project brought together strong expertise in AVs, urban planning, spatial analysis and transportation simulation from academic and research institutions, with planners and policymakers in multiple government agencies. This project followed a transdisciplinary action research approach [39], evolving with inputs from the implementing planners and policymakers, helping to sharpen the project focus and grounding it in reality.

The initial project design followed a standard approach, where several design options were to be explored in a study area, two of which would be selected for detailed high-resolution island-wide simulation using a state-of-the-art agent—it intends to be prescriptive while informed by prediction-based simulation platform, MATSim [40]. The performance of the simulated scenarios would be compared with the baseline simulation (with no intervention) to select one optimal solution. However, when put into practice, this approach faced several roadblocks.

**Figure 2.**
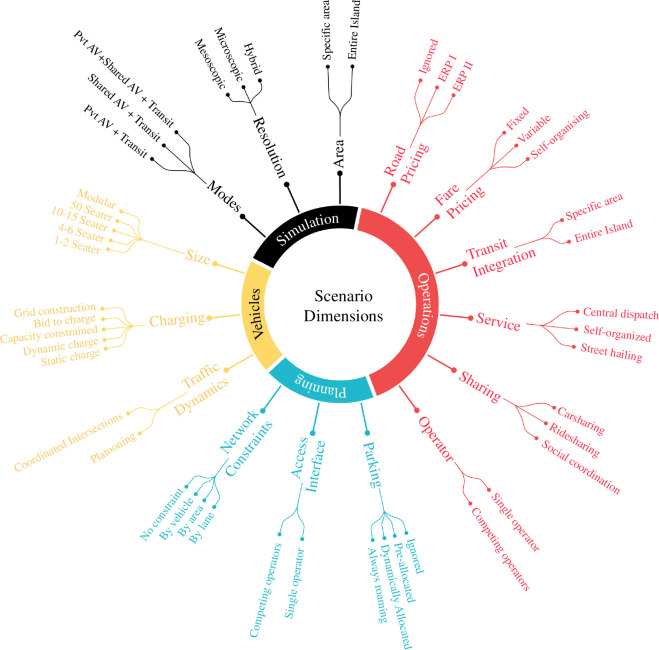
Possible scenario dimensions to be considered. Source: Adopted from Trinh *et al*. [41].

The research team and project stakeholders could not reach a consensus on the selection of the initial design option set to be simulated, given the novelty of these technologies and uncertainty about the impacts of interventions. Up to 15 possible scenario dimensions were identified in early project meetings (see figure 2), which alone would generate 15 million plausible models through a combinatorial explosion. At the same time, as the construction of the baseline model began, it became clear that the size and simulation runtimes would be too large to test several design options. An alternative ‘design experiment’ approach was proposed instead to facilitate the selection of options to be simulated on MATSim.

Instead of constructing high-detail simulation models for a few selected options, several low-detail heuristic models can be constructed, carefully designed around a question of interest. An ensemble of such coarse models based on a single question of interest is defined as a ‘design experiment’ [42]. This model ensemble is purpose-built, designed to answer specific questions under scrutiny, instead of representing the entire system. Therefore the models are seen as heuristic planning tools, not expected to be accurate or predictive. With a sufficiently diverse ensemble of models, certain system properties can be inferred, enabling quantitative comparison of design strategies, cause-and-effect mechanisms and the trade-offs involved, to inform the final design synthesis. Such low-detail heuristic transport models enable an iterative cycle of design and transport simulation, where they can mutually inform each other.

### (a) Selection of modelling platform

The complex interplay between urban systems and the unpredictable behaviours of populations within them calls for a modelling paradigm adept at capturing emergent phenomena and plausible behaviour of the travelling population in response to such emergence. The modeller should also be able to trace back emergent phenomena to specific entities within the model that correspond directly to real-world counterparts. In this regard, agent-based simulation is a preferred modelling paradigm, which, unlike differential equations or system dynamics models, is uniquely suited to capturing the bottom–up interactions that give rise to significant system-wide behaviours and phenomena. Their challenge, however, lies in the potentially time-consuming nature of simulating and feeding data into these models.

Agent-based models become increasingly unwieldy in the pursuit of higher accuracy. The modeller must translate design inputs into a model specification and manage the simulation process to ensure quick turnaround times while capturing critical emergent phenomena that are vital for informing design decisions. Open-source technology is advocated here, as it offers maximum flexibility in modifying the simulation to introduce new behaviours or streamline processes. Furthermore, an open-source approach encourages collaboration, leveraging a shared code base for collective advancements in the field.

Agent-based simulations in MATSim were used in this project to assess different design options through design experiments. MATSim is particularly useful for this analysis since it is an activity-based, extendable, multi-agent simulation framework, which allows different design configurations to be simulated [43]. MATSim features a co-evolutionary solution approach, where agents adapt to emergent conditions over successive iterations. Since MATSim is designed for large-scale scenarios, it adopts the computationally efficient and simplified queue-based approach (QSim). This is much quicker than detailed modelling of vehicle movement found in other truly agent-based microsimulation approaches. However, as the scale and complexity increase, especially in computationally expensive modules that support demand-responsive transport (DRT), the model runtimes can be very long.

Simulation models for heuristic planning must exhibit the following three essential properties [42]:

1. Agility: The simulation run times must be short to keep pace with the iterative design process, allowing testing of multiple design scenarios at early stages, with very coarse data inputs.
2. Modularity: While the model resolution needs to be reduced to achieve agility, some features may also need to be modelled in high resolution, depending on the question of interest. For example, if the design of intersections is of key concern for the intervention under study, the definition of intersections needs to be more nuanced in the model. For this reason, the modelling platform should be modular.
3. Intelligibility: If the decision-making is to be informed by simulation models at early stages, the model should be intelligible, making cause-and-effect dynamics clear and explicit, preferably in a visual format intelligible for a non-technical audience.

The project team developed an agile (albeit coarser) version of MATSim with a visual interface, ‘Sketch MATSim’, incorporating all these essential properties to some extent. Sketch MATSim is an experimental tool used in this project, with the ability to generate plausible travel demand instantly from a massing of buildings, based on their land use agglomeration and square footage as well as basic accessibility. The rapid agent-based travel demand generation model is trained against the country’s existing building stock with aggregated mobile phone data. Using this approach, the trips produced and attracted by the building stock, based purely on building attributes, agglomeration and basic accessibility, will more-or-less reproduce the aggregate 24 h mobile phone origin-destination matrix. When the model is run against a new set of buildings in an entirely new urban area, it will create a travel demand that would be plausible, at the very least, for the given urban design configuration [44].

### (b) Proposed new iterative process

**Figure 3.**
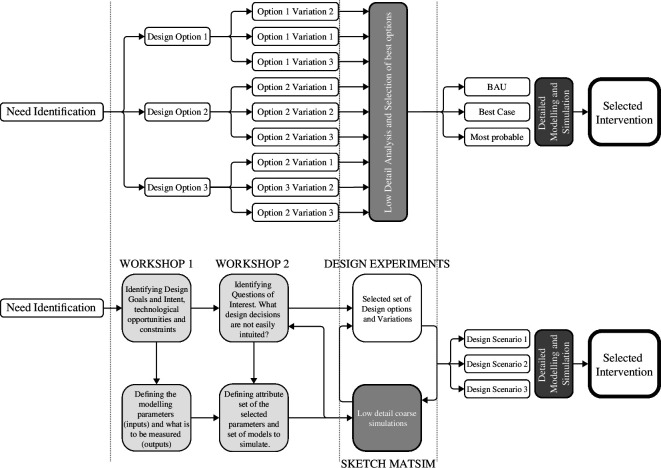
Proposed iterative workflow with heuristic models (below), compared to traditional workflow (above).

The development of Sketch MATSim facilitated an iterative process of decision-making in the design of transport infrastructure for AV deployment in Singapore. The core of the proposed approach is heuristic modelling informed by design optioning and vice versa, in an iterative loop, where the inputs (what is to be simulated) and outputs (what is to be measured) in the simulation model are co-designed with the stakeholders, as shown in figure 3.

In the first instance, we need to establish consensus among the stakeholders on the design goals, intents, opportunities and constraints. In this project, the stakeholders included the extended research team and representatives from government agencies (local policy and planning agencies responsible for transportation, housing and urban development). A design workshop was used as the primary method to elicit responses. The results of this workshop informed the parameters to be modelled in the heuristic models—the level of detail of the simulation model (what is abstracted out and what is represented in high detail) and the rules and dynamics of the model—as well as the performance measures, i.e. what needs to be measured to assess the design options.

A second workshop was conducted to narrow down the key questions of interest to be investigated through modelling and simulation. This workshop was designed to unpack only those key questions of interest that are not easily answered through static spatial analysis, precedents or normative design principles.

Based on these workshops, ensembles of purpose-built heuristic models were constructed around specific questions of interest, comprising a ‘design experiment’, revealing the structural trade-offs and cause-and-effect dynamics of design strategies through an iterative design and simulation process. Co-designing the simulation models not only helped narrow down the solution space but also improved the simulation models by fine-tuning assumptions and underlying dynamics. The outcomes selected for more detailed simulation at a large scale using MATSim following this process proved to be more robust and useful.

## 4. Workshop 1: identifying goals, intent and measures

The first workshop was attended by 35 participants, which included the project research team and government stakeholders. The workshop facilitators established a baseline understanding of the opportunities and constraints of AVs and their impacts on urban form and transport supply. Following this, the participants broke out into three groups to discuss what ‘AV’ means in their given context.

**Figure 4.**
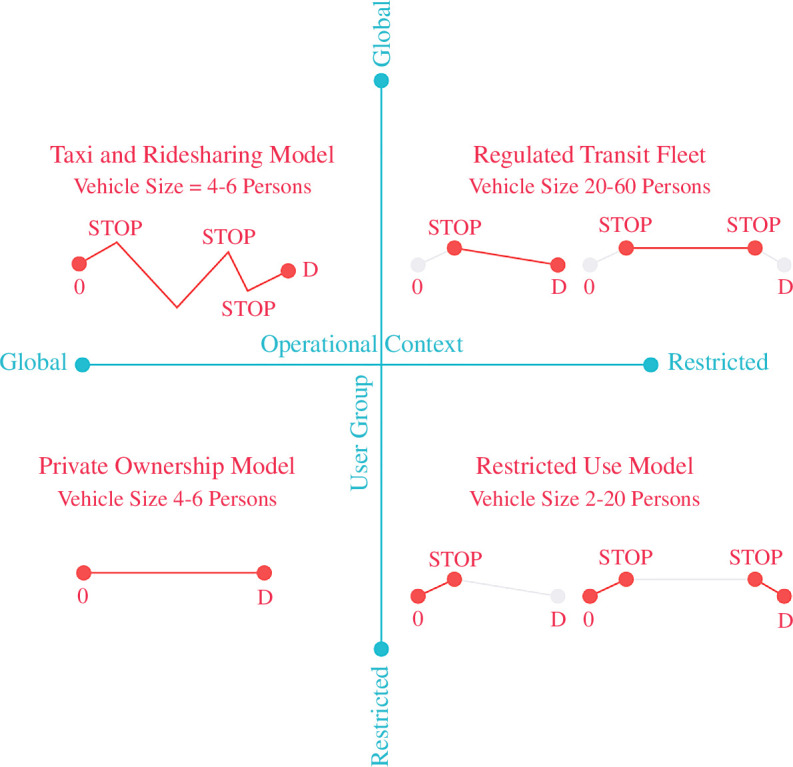
Matrix of four operational models for AVs.

Participants were asked to map by whom and where AVs should be operated and used, on a linear scale, from global access to restricted access. The first axis is the operational context. A restricted operational context implies that AV use is restricted in space, either in an ‘AV-only’ zone or lanes/streets. This could be because of limitations in technology or intentionally restrictive policy. The second axis is the user group. The user group could be restricted (e.g. by place of residence or ability) or any user could be allowed to access an AV globally. The latter is possible when AVs operate as shared taxis or buses. The two axes give four extreme operational models—the private ownership model, the restricted-use model, the regulated transit fleet model and the taxi/ridesharing model (see figure 4).

***Private ownership model***
In this model, any car buyer can choose between human-driven or automated cars to operate in all areas unrestricted. AVs are accessible to only those who can afford to buy a vehicle. This would result in an upgraded experience for the existing car driver, at the risk of increasing overall car ownership, as vehicles become more versatile and attractive.
***Taxi and ridesharing model***
In this model, there are no personally owned AVs. Instead, all AVs are shared and operate as taxis or dynamically routed transit unrestricted on a city-wide scale. Users can choose from a range of options between the more expensive private taxis and cheaper shared vehicles. Such a system could be run by competing enterprises or a single operator, guaranteeing maximum efficiency but risking market monopoly.
***Government regulated fleet***
In this model, AVs operate as large vehicles in a government-managed and/or operated fleet of fixed-route buses. The bus routes are centrally planned, and the bus lanes are designed to be segregated from normal traffic, like a bus rapid transit system. The transit operator can exert more control over mode share distribution in this model.
***Restricted-use model***
In this model, AVs can only operate in a restricted area pre-fitted for operation. This may be due to a lack of trust in technology, or as a mechanism to control overall vehicle kilometres travelled (VKT). AV use could be restricted to closed areas, like a college campus.

Each model has its advantages and disadvantages, and no single model is a ‘best fit’. A combination of all types of services needs to be implemented for different urban contexts and user groups. Three types of urban contexts were selected for discussion—(1) residential new towns (2), central business districts and (3) mixed-use districts. Each group was assigned one type of development and asked to examine the operational models that would suit the development type.

### (a) Workshop outputs

**Figure 5.**
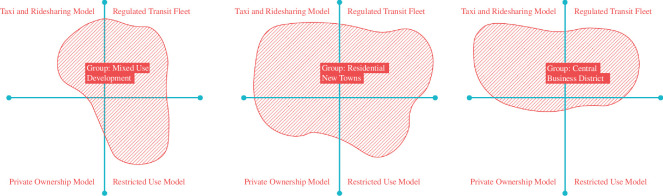
AV operating models for three urban contexts.

There was unanimous agreement between the three groups regarding the need to move towards a ‘car-lite’ future in Singapore, reduce reliance on private cars and encourage public transport, cycling, walking and car-sharing services. Accordingly, all groups favoured the ‘Regulated Transit Fleet’ model over the rest, followed by the taxi/ridesharing model and, the restricted-use model for specific purposes, as shown in figure 5. The private ownership model was least favoured by all groups. There was a fear that the taxi and ridesharing model may affect the mode share of the regular transit service. Mass rapid transit (MRT) of Singapore is considered the backbone of the transport network and all participants agreed that the new services must be designed to support it. Thus, taxis and ridesharing were only allowed in restricted areas to serve first- and last-mile trips.

A combination of taxi, ridesharing and fixed-route transit was selected in the CBD development type. Streets in business districts are utilized highly unevenly, between weekdays and weekends and between inflow and outflow directions during peak hours. A dynamically adaptive street was proposed where the lane directions would be demand-responsive. During off-peak hours, the lanes could be blocked out entirely and used for other purposes. These proposals implicitly assume that connected AVs will dynamically respond to geo-specific governing policies to determine their behaviour.

There was no consensus on an operating model that might be most appropriate for a residential New Town. Instead, it was agreed that a spectrum of services across all quadrants was needed. While private vehicles and taxis provide a direct trip with no transfers, thus improving the quality of the trip, a regulated fleet is more space efficient. The participants believed that both types of services were necessary. A restricted-use model was found to be more suitable for a new town, where the fleet could be owned by a residential association, to serve first/last-mile trips to public transit. The residential new town group suggested that a fleet of small vehicles could address the problem of fleet efficiency, by serving full trips as a taxi or coupling together in a platoon to serve long-distance trips. The modularity of vehicles also addresses the problem of empty large vehicles during off-peak hours, which is common in residential neighbourhoods.

As the groups debated specific operational models for AVs for their given urban development type, some infrastructural concerns arising from AV deployment on their sites emerged, primarily regarding street and parking capacities. Can existing streets accommodate the expanded AV transit fleet? Some participants proposed dynamic use streets for the business district as discussed before. The opinion was divided on the shared use of streets, with mixed traffic of AVs, pedestrians, cyclists and personal mobility devices. While some saw vehicle automation as an opportunity to create more lively streetscapes, others were sceptical about the feasibility of such a design from the point of view of technological competence.

Parking was another point of contention in the groups. An ‘always cruising’ strategy was suggested instead of a stationary parking lot. A mixed-use neighbourhood has internal transport flows in both directions, leading to the possibility that an SAV may never need to park. An SAV could cruise at an energy-efficient speed and position itself in an area where demand is anticipated. The upper floors of existing parking structures could be retrofitted for vehicle charging, repair and maintenance purposes. However, there were fears that this strategy would lead to service instability and an increase in VKT, often used as a proxy for vehicle-based emissions.

Pick-up and drop-off (PUDO) areas for a large fleet of small, shared vehicles were raised concerns. Participants were unsure about the number and size of PUDO points needed. Some participants suggested compulsory provision of PUDO points in every residential block, while others questioned if there was enough capacity to accommodate them. It was also recognized that merely providing a network for pedestrians and cyclists was insufficient to support active mobility. Provision of more activity opportunities was suggested to create a more attractive environment to walk and cycle.

### (b) Implications for modelling

Two primary implications of this workshop for modelling were the definition of mode and urban context of operation. All participants agreed on placing the focus on a ‘car-lite’ future city, rather than an ‘AV-ready’ city. The purpose of the AV would be to support existing public transport systems and active mobility. A taxi and ridesharing model integrated with a fixed transit fleet was prioritized. Consequently, design experiments and simulations would be constructed with a fleet of SAVs and DRT system, integrated with scheduled services in a mobility-as-a-service configuration. Residential New Towns were identified as the ideal urban context of operation for the DRT system. Eighty per cent of Singapore’s resident population lives in New Towns [45], which are served by at least one MRT station. DRT is envisioned as a supplement to the MRT, ideally suited to improve the accessibility to transit hubs in New Towns. This could be implemented by restricting the area of use of DRT within the New Town.

## 5. Workshop 2: exploring the design strategies

The second workshop was designed to brainstorm the design implications of the four AV operational models discussed before, with 40 participants from the research team and government stakeholders. A 2 × 1.5 km large fictional new town site was provided as a base to test design strategies for a new residential town where all operating vehicles are automated. The selection of design strategies was informed by previous workshops, literature reviews and an international horizon scan of design strategies for AVs. The participants were asked to design a new AV-integrated residential town on this site. Since this workshop heavily relied on design as a method of inquiry, each group was provided with a draughtsperson to support them.

**Figure 6.**
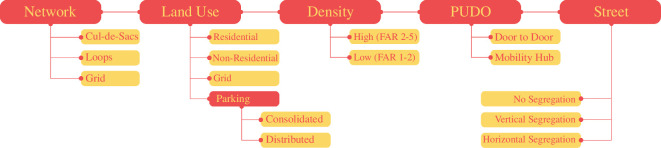
Layers and templates given to participants in workshop 2.

A ‘layer and template’ method was used to produce designs in each group as shown in figure 6. Five ‘layers’ of urban form that influence transport flows were identified—Network, Land Use and Parking, Development Intensity (or Density), PUDO and Street Profile. A set of design options or ‘templates’ for each layer was used. Participants were asked to assemble their designs layer by layer, using this set of Singapore-specific templates. This systematized method eased design production for the participants who were policymakers and researchers, without a background in spatial design. The Layers and Templates were organized as follows.

***Network***
Three grid templates were offered—a disconnected cul-de-sac dominant grid, a reasonably connected loop-based grid, and a highly connected fine-grained grid.
***Land use and parking***
Four main sub-layers form the land use—residential, which are the origins of flows, non-residential (retail and offices), which are the destinations of flows, and green spaces, which may account for recreational/off-peak flows. The fourth layer, parking, has two additional templates—large consolidated parking lots or smaller distributed parking structures, similar to the current convention.
***Density***
While land use determines the direction of transport flow, density determines the intensity of flow. Two templates were provided for this layer—high density (floor area ratio 2–5) and low density (floor area ratio 1–2).
***PUDO***
Two templates were provided for pick-up/drop-off activity—a dedicated PUDO for door-to-door service and an integrated mobility hub. The spacing of the PUDOs could be more frequent in the former case, and less in the latter.
***Street***
The street layer addresses how AVs may influence the active mobility experience and the templates determine how and to what extent non-automated users will be segregated from the AVs. Three templates were provided—no segregation at all (mixed traffic), vertical segregation (through bridges and underground tunnels) and horizontal segregation (either through fences and buffers between lanes or complete separation of networks).

Participants were asked to develop these five layers for the site provided, using one of the quadrants in the operational model matrix (figure 4) as the dominant model on their site. Once participants assembled all five layers through overlays, annotated maps, sketches and drawings, they were shared in an open forum. The points of consensus and debate were recorded by the note-takers during the discussion, which directly informed the ensemble of heuristic models.

### (a) Workshop outputs

**Figure 7.**
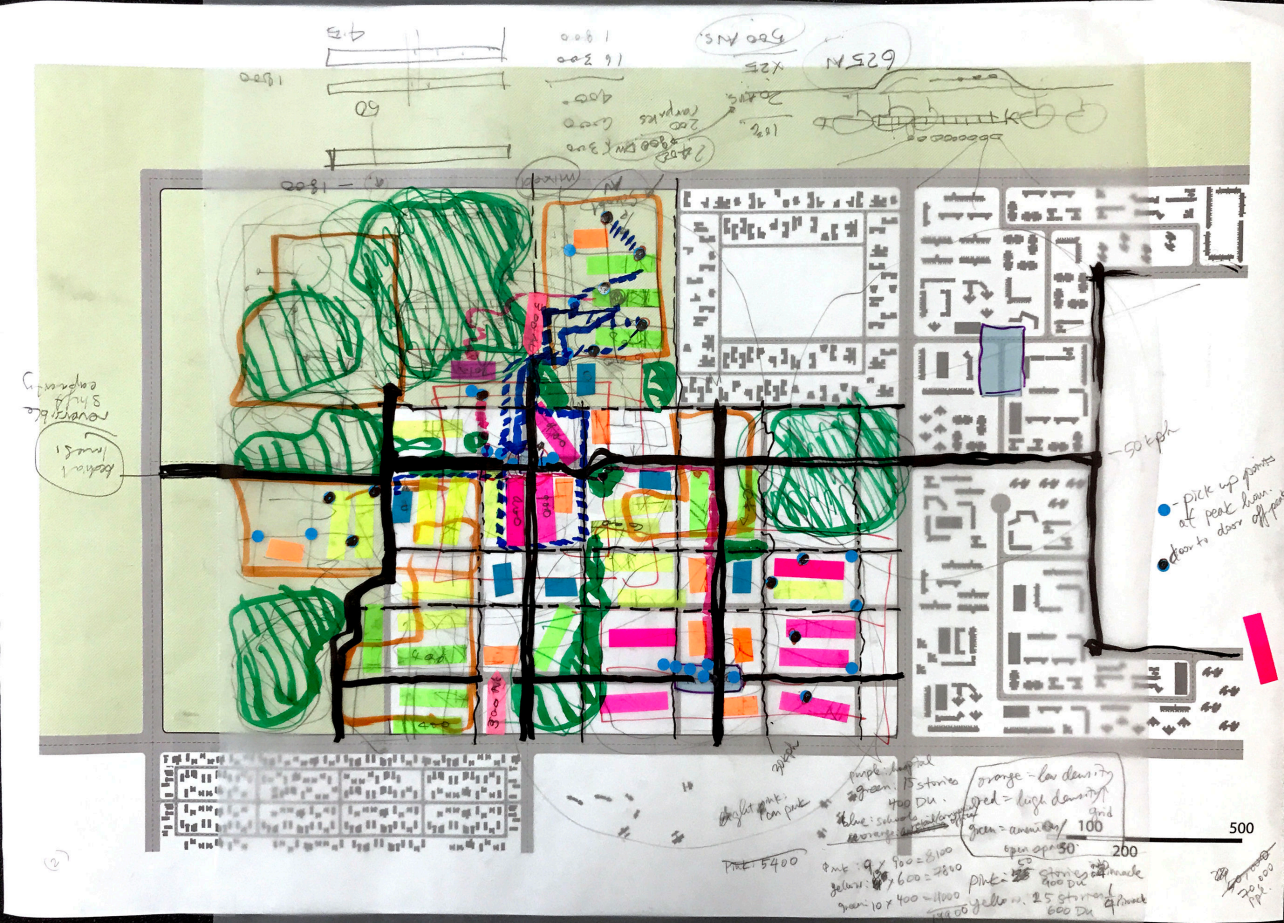
Example of one assembled set of layers with provided templates.

The four groups presented four design proposals by assembling the design layer by layer, using the set of templates provided (as an example, see figure 7). All four designs had certain commonalities and points of contention. There was general agreement across all groups on vehicle types, density and integration of active mobility infrastructure. Smaller agile vehicles were preferred, which would operate as DRT. The density fluctuated between medium to high, given the need to accommodate new development while maintaining existing green areas and maximizing the efficiency of public transit and ridesharing systems. All groups also provided a dedicated network for active mobility, which either coincided with the traffic network while separating from it through a physical barrier, or was designed as an entirely separate network layer.

The main points of debate were the design of intersections, PUDO, parking and network. While some participants preferred complete physical separation between AVs and other traffic at intersections, others were sceptical about the impact of this strategy on walkability. There was also a lack of clarity around the optimal size and location of PUDO points and parking. For example, while some participants advocated building underground parking structures, it was difficult to determine its feasibility, if the total vehicle stock is unknown. The selection of network topology design was contingent on the type of operating model, land use distribution and intended mode share. Each group presented arguments for and against all three network types based on different transportation goals.

### (b) Implications for modelling

Following the two workshops, several variables were debated and confirmed through several targeted meetings. The model definition became more specific and manageable as assumptions were made regarding the demographic, environmental, technological and operational conditions, reducing the number of variables. The consensus-building process within the constraints of the software technology was long and contentious and directly informed the formulation of design experiments.

There was general agreement among the stakeholder group on the vehicle types and development density to be simulated and the goal to prioritize active mobility infrastructure. However, some points of contention were identified where no decision could be made. Four of these were specifically related to urban design strategies, summarized in the following four questions of interest:

1. How can the design of the street network support public transit and shared modes?
2. In the event of the end of private car ownership and total vehicle automation, how will we access transport options?
3. What will be the new requirements for parking infrastructure?
4. If AVs operate most efficiently when segregated environments, who gets priority on streets and intersections?

These questions informed the four iterative design experiments to be modelled and assessed using Sketch MATSim.

## 6. Iterative design and simulation with Sketch MATSim

The questions of interest identified through the workshop provide the parameters that need to be modelled and tested through four different design experiments—network, PUDO, parking and intersections. For each experiment, three extreme design strategies were modelled and simulated in Sketch MATSim to understand the cause-and-effect dynamics underlying the design decisions. For example, in the Network experiment, while all other base conditions remain the same, we want to investigate how different network designs impact transport flows, with shared and connected AVs.

A low-detail parameterized base model was constructed with a narrowed scope, mimicking only the essential properties of the site relevant to the research questions. This parametrized model was modular enough to be modified for different design experiments. The following three extreme network types were tested for the Network experiment: the less connected *Loops* network, the well-connected *Grid* network and the *Superblock* with high-speed peripheral roads and restricted low-speed internal roads. Each of these networks may offer some benefits or pose some threats, which are impossible to determine intuitively.

The three models were simulated in Sketch MATSim to understand how network design impacts the overall goal of sustainable urban development in Singapore and sub-goals of space efficiency, low carbon emissions, improvements in walkability, smooth traffic flow and better access to public transit (for a detailed analysis of the Network experiment, refer to Maheshwari *et al*. [42]). The understanding of the use of the network over time also helped to deduce where peaks and bottlenecks are created, allowing the designer to modify the plan accordingly. The agility of Sketch MATSim enabled the designer and modeller to make small tweaks in the models and observe the changes to understand the underlying dynamics. The results from the four design experiments informed the final scenarios to be simulated in high detail island-wide and compared with the baseline conditions.

## 7. Reflections

The iterative process of decision-making in planning for new transport technologies, although time-consuming and challenging, provided new knowledge and understanding, not only for improvements in design proposals and simulation models but also in communicating the design intent and modelling limits to policymakers.

### (a) Challenges

The consensus-building process to arrive at a single set of assumptions and performance measures that met the requirements of all stakeholders within the technical and context-specific constraints was long-drawn and contentious. Through the span of a research project, unexpected external developments, such as a change in political direction or a global pandemic, can reverse previously agreed-upon decisions. Therefore, building more agile models and seamless workflows between the design and simulation is key to enabling responsiveness to unforeseen conditions. Co-designing transport models as heuristic planning tools is a deliberate effort to build agility into the research process.

A second challenge is addressing the biases built into a co-design process. Since researchers collectively decide, in consultation with stakeholders, what details need to be represented in the model, some voices may remain unheard, and personal biases of the stakeholder group may be overly represented. For example, land use distribution is a critical factor influencing transport flows. However, it was not included in the design experiments since land use provision is relatively stable in Singapore, with most of the residential land being publicly held by a central authority. Since the intent of a heuristic model is to provide a structural understanding and not an accurate ‘solution’, more iterations can be designed to include new stakeholders and emerging needs over time to create an ‘evolving genealogy of models’ [35].

A final challenge was reconciling urban design and simulation workflows that tend to be very different. For example, for every design alteration, however minor, a new demand description and network was needed which can be time-consuming. The larger the area of simulation (or the number of agents), and the higher the level of complexity (for example, using DRT instead of a fixed bus network), the longer the run time of the simulation, which cannot match the quick iterative pace of design. Additionally, design elements of interest, such as parking design, may not be explicitly manipulable inputs for the simulation. Given these constraints of model set-up time, simulation run time and low spatial granularity, the integration of design and simulation can be severely restricted. Sketch MATSim allowed quick evaluation of design scenarios at the required spatial granularity. However, it must be acknowledged that significant work remains to hone the fine balance between model detail and agility.

### (b) Opportunities

The collaborative co-design process grounded the research in real-world practice and helped manage the scope of modelling and simulation. An important opportunity afforded by this project was the ability to build upon the substantial expertise in agent-based transport simulation that Singapore has developed over the past 15 years. Through strategic research initiatives hosted at the National Research Foundation’s Campus for Research Excellence and Technological Enterprise (CREATE), international universities have collaborated with local institutions, significantly advancing the field of transport simulation. Projects such as the Singapore-MIT Alliance for Research and Technology’s development of SimMobility [46], Carlo Ratti’s Senseable City Lab at MIT utilizing real-time data to analyse urban mobility patterns [47] and the Technical University of Munich’s creation of CityMoS focused on urban mobility simulation [48] have contributed to a rich foundation of knowledge. The National University of Singapore has also contributed expertise in electric vehicle planning and charge location optimization, integrating agent-based modelling into their research [49]. This depth of expertise has been transferred to the Land Transport Authority, which now employs simulation technology across nearly all of its planning operations [50,51]. Our work benefits from this rich landscape of knowledge and experience, enabling us to enhance the iterative integration of urban design and transport simulation through our co-designed heuristic modelling approach.

This approach also provided new opportunities for knowledge transfer, involving the decision-makers in the modelling process as co-designers and allowing them to ‘rehearse the future’ [52]. An exhaustive list of uncertainties, drivers and assumptions was gradually revealed through a co-learning process between the policymakers and researchers, rather than having results produced by a black-box model reported at the end of a typical research project.

An essential part of any applied research process in urban planning and design is the communication of design proposals and research findings to the stakeholders. The knowledge produced needs to be translated between disciplines, as well as between researchers who are subject area experts, and policymakers, who tend to be generalists. There are plenty of software and tools available today that facilitate such knowledge transfer and sharing through data visualization and interpretation, nonetheless, obtaining a thorough understanding of the dynamics in a complex system can be challenging.

Design thinking methods were useful for facilitating collaboration in this project. The design workshop format allowed the imagination to run freely, giving material expression to theoretical insights, grounding them in reality and providing a platform for collaborative speculation [53]. In addition to gaining insights from participants, the design workshops were also an effective tool for communicating research findings to stakeholders, rather than a report or presentation. Working with visual scenarios that represented research findings in a physical space was effective in eliciting a response and sparking debate.

Another innovative aspect of this research was the use of multi-agent modelling in conjunction with urban design to build insights. Agent-based simulations added a temporal perspective, which is mostly lacking in traditional urban design methods. Emergent effects, such as the trickle-down effect of walkability improvements for all transport modes, became clearer through the models. By tightly knitting together urban design and simulation processes, not only were the design proposals better informed, but the description of the simulation models also improved.

The design inputs streamlined the model description to target practical applications, pruning away superfluous details. The graphic user interface of Sketch MATSim allowed designers to interact with the data and reconcile design and simulation models in one space. Multiple simulation cycles with small tweaks in design exposed weaknesses and bugs in the model. This offered new insights to the modeller, who under normal circumstances would not iterate so regularly. Both designer and modeller develop a joint understanding and intuition for the levers of control over the future mobility system, which gradually reveals itself over repeated iterations.

### (c) Limitations

Sketch MATSim is a product under development, and considerable technical expertise is still required to build the base model for simulation and experimentation. For each new question of interest, the definition of a new experiment and the development of relevant new modules is required. Demand estimation requires further work, since it is a critical aspect of planning and design, and is already challenging in MATSim (where demand is modelled exogenously). In this project, demand was estimated using a rapid data-driven process, such that changes in the urban design/land use configuration are immediately translated into a functional travel demand description and able to be run in minutes for comparison against previous baselines [44].

Model validation and calibration concerns have already been raised for consolidative modelling [54,55] and are exacerbated in heuristic models. There’s a trade-off between the level of detail in the model and the degree of significance to which impacts can be attributed to a particular design intervention in the model. However, since the goal of the models is to obtain a structural result, validation is not of key concern [35]. The pursuit of perfection should not hinder the good. By ensuring that the physics of the system in the agent-based model are plausible, comparative analysis can be performed and intuitive insights for the system and its parameter space for feasible operation can be explored even if there is no certainty that they will be in exact agreement with the future system.

Several assumptions were made to simplify the simulations and reduce runtimes. The subjects of many of these assumptions merit a targeted study, given their important role in influencing transport flows. These include the pricing strategy, land use and density variations, cost of building new infrastructure, transit service reliability and public acceptance of new technologies. Some types of urban transport flows are also excluded from the simulation model, such as freight and service traffic, which accounts for a large proportion of transport flows in the city.

## 8. Future work

Sketch MATSim requires improvement in its travel demand estimation process to make the process transferable across various contexts and levels of data availability. The contributions to the code base of the MATSim project continue to provide an increasing number of future mobility options that can potentially be tested. There is also the potential to train a machine learning model over the various dimensions of the design experiment space and provide more rapid explorations and discovery of interesting combinations of urban design and mobility system configurations [56].

Heuristic models are used in this research as a mediating object to test, modify and change according to the different needs and preferences of the stakeholders involved. The insights from the application of complex analytical models cannot be used to predict the future, but through this research, were found to be useful tools for building consensus and enabling social learning.

## Data Availability

This article has no additional data.
